# Understanding the path to ketamine-assisted group therapy for firefighters diagnosed with post-traumatic stress disorder: a qualitative study

**DOI:** 10.1192/j.eurpsy.2025.1969

**Published:** 2025-08-26

**Authors:** T. Walia, V. Tsang, K. Sattler

**Affiliations:** 1 Biology, McMaster University, Hamilton; 2 Medicine; 3 University of British Columbia, Vancouver, Canada

## Abstract

**Introduction:**

Firefighters face routine occupational risks and high-stress environments. Accordingly, 96.4% are exposed to a potentially traumatizing event throughout their career (Nazari *et al*. Work 2020; 67(1):215-222). This is accompanied by high rates of trauma-related mental illnesses (TRMI) such as PTSD. First-line treatments of TRMI remain limited. A novel treatment that has emerged, Ketamine-Assisted Therapy (KAT) has shown meaningful improvements in PTSD treatment (Ragnhildstveit *et al.*Ther Adv Psychopharmacol 2023; 6:13).

**Objectives:**

The primary objective was to synthesize prevalent themes among participants, shedding light on their journeys to accessing KAT as well as highlighting internal motivations and societal barriers that exist.

**Methods:**

In this study, there are six working firefighters, diagnosed with TRMI. They have enrolled in but not begun the 12-week Roots to Thrive (RTT) KAT program. Interviews were conducted individually and transcribed for further analysis. Data from the interview responses was analyzed by two independent reviewers and coded to create main themes of focus. The inconsistencies were managed by the third senior reviewer.

**Results:**

The major themes prevalent among participants include social stigma, self-advocacy/education, logistical barriers and feeling stuck with previous treatments.Throughout the interviews, participants indicated that the use of ketamine and their mental illness diagnosis were stigmatized. Some kept their treatment a secret from family members while others reported telling very few colleagues about their PTSD. In response to traumatic events at work, one participant explained that the prevalent attitude was “Suck it up. Move on.” The lack of administrative support prompted several participants to teach themselves about psychedelics and mental health in order learn about treatment possibilities. Logistical barriers included barriers to physically commuting, paying for and having time away for the KAT sessions. They pointed out that aside from the price of the treatment itself, paying for cabs and ferries adds to the costs. Each of the participants detailed their journey with trauma and mental illness before KAT. A common sentiment expressed was that they couldn’t cope anymore with symptoms like night terrors and extreme anxiety. Participants discussed a gradual build-up and burnout, as one explained, “You continually are always going to other trauma calls, so it kind of builds up layer, layer, layer after layer.” A reoccuring theme in their experiences was trying different treatment modalities (EMT, EMDR and CBT) with little permanent success.

**Conclusions:**

Greater funding, awareness and administrative support is needed for firefighters diagnosed with PTSD to successfully receive and undergo treatment.

**Disclosure of Interest:**

None Declared

